# Program Directors’ Selection Criteria for General Surgery and Internal Medicine Residency Programs: A Cross-Sectional Study

**DOI:** 10.7759/cureus.31218

**Published:** 2022-11-07

**Authors:** Mohammad I Almatrafi, Nardeen I Alsweed, Afrah A Alharthi, Abdulaziz S Aljuaid, Radwan J Samkari, Mohannad T Hemdi, Rani Alsairafi

**Affiliations:** 1 College of Medicine and Surgery, Umm Al-Qura University, Makkah, SAU; 2 College of Medicine and Surgery, Princess Nourah Bint Abdul Rahman University, Riyadh, SAU; 3 College of Medicine and Surgery, Taif University, Taif, SAU; 4 Department of Surgery, Faculty of Medicine, Umm Al-Qura University, Makkah, SAU

**Keywords:** internal medicine, general surgery, applicant selection, residency, program directors

## Abstract

Background and aims

General surgery is a specialty that calls for a variety of abilities such as strong hand-eye coordination, the ability to function well under pressure, and the ability to make quick, informed decisions. On the other hand, internal medicine focuses on diagnosing, treating, and preventing adult non-surgical disorders. The present study aims to investigate the commonly used selection criteria employed by program directors (PDs) for general surgery and internal medicine residency programs. It also identifies how PDs value those criteria in the western and central regions of Saudi Arabia.

Methods

A retrospective cross-sectional study was conducted using a questionnaire adopted from the literature and modified following expert opinion. It was distributed to PDs in both the central and the western regions of Saudi Arabia.

Results

In total, 34 PDs completed the questionnaire, of which 32 (94.1%) were men. The mean age of participants was 42.53 ± 5.05 years; 21 (61.8%) PDs were general surgeons, and 47.1% were from the Jeddah region.

Conclusion

The study showed that the most selected criterion for both specialties was communication skills followed by clinical rotation in the same hospital; leadership skills were also highly considered by PDs.

## Introduction

General surgery (GS) is a specialty that calls for a variety of abilities such as strong hand-eye coordination, the ability to function well under pressure, and the ability to make quick, informed decisions [1]. One of the foundational educational initiatives of the Saudi Commission for Health Specialties (SCFHS) was the Saudi Board of General Surgery, which was established in 1995. Approximately, 600 people apply for the five-year program annually, with only 140 applications being approved. Seven of the 26 Saudi hospitals that offer GS training programs are located in Makkah, Jeddah, and Al-Taif [1,2]. Internal medicine (INTMED) focuses on the diagnosis, treatment, and prevention of adult non-surgical disorders. In 1995, the SCFHS created the INTMED Saudi board, which consists of a two-year subspecialty fellowship program after a four-year residency training program. It is considered as a highly competitive specialty as the number of applicants is up to 1000 every year and only 350 of them get accepted [1,3].

The most crucial evaluation criteria for approval must be identified because the number of candidates is continuously increasing [4]. Hence, the future of these two specialties depends upon the successful selection of applicants for residency programs by following the most accurate criteria [5,6].

According to research, the assessment of surgical skills is the primary criterion for admission to residency programs in Ireland, but this criterion is not used in institutions in the United States [7]. Additionally, studies have shown that the main criteria for acceptance into GS residency programs are the applicant’s interest in the field, knowledge of the field, and participation in departmental rotations followed by their contribution to clinical research [8,9]. For INTMED, the PANEL style, which includes a balanced and uniform assessment method, is used to evaluate applicants during interviews [6,10]. This tool has numerous interviewers, including the program director (PD)/associate PD, a member of the faculty, and the chief of residents, who evaluate the applicant based on multiple subjective and objective factors [6].

However, few studies consider PDs’ criteria for selecting applicants to GS and INTMED programs in Saudi Arabia. Thus, the aim of this study is to investigate the PDs’ commonly used selection criteria for GS and INTMED residency programs. It also aims to identify how those criteria are valued by PDs in the western and central regions of Saudi Arabia.

## Materials and methods

Study design, setting, and time

This is a cross-sectional study, which was carried out in the western and central regions of Saudi Arabia in July and August 2022.

Study population

The target sample was GS and INTMED PDs in these regions. The inclusion criteria were GS and INTMED PDs working in the western region or central region of Saudi Arabia. The exclusion criteria were other specialty PDs, registrars, residents, and interns.

Data collection

Data were collected by the researchers using an electronic self-administrated questionnaire. The first section of the questionnaire included items about PDs’ personal information. The second section included their rating of the importance of different selection criteria for GS and INTMED residency programs (e.g., components of applicants’ CV, Saudi Medical Licensing Examination [SMLE] score, recommendation letter, and clinical rotation).

Ethical considerations

Ethical approval for the study was obtained from the Ethics Committee of Umm Al-Qura University (HAPO-02-K-012-2022-02-945).

Data analysis

Data were statistically analyzed using SPSS version 26 (IBM Corp., Armonk, NY). To investigate the association between the variables, the chi-squared test (χ^2^) was applied to the qualitative data expressed as numbers and percentages. The association between the quantitative parametric variables expressed as mean and standard deviation (mean ± SD) was examined using one-way ANOVA. A p-value of less than 0.05 was regarded as statistically significant.

## Results

As shown in Table 1, 34 of 50 PDs completed the study questionnaire. The mean age was 42.53 ± 5.05 years. Most of the respondents were men (32 [94.1%]), and 21 (61.8%) were general surgeons. The majority were from Jeddah (16 [47.1%]). In total, 28 (82.4%) PDs rated communication skills as very important and 20 (58.8%) rated the SMLE score as very important, followed by the applicants’ clinical skills and knowledge (15 [44.1%]).

**Table 1:** Distribution of participants by their demographic characteristics (N = 34)

Variables		No. (%)
Age		42.53 ± 5.05
Gender	Female	2 (5.9)
	Male	32 (94.1)
Specialty	General surgery	21(61.8)
	Internal medicine	13 (38.2)
Region	Jeddah	16 (47.1)
	Makkah	8 (23.5)
	Riyadh	4 (11.8)
	Al-Taif	6 (17.6)

**Figure 1:**
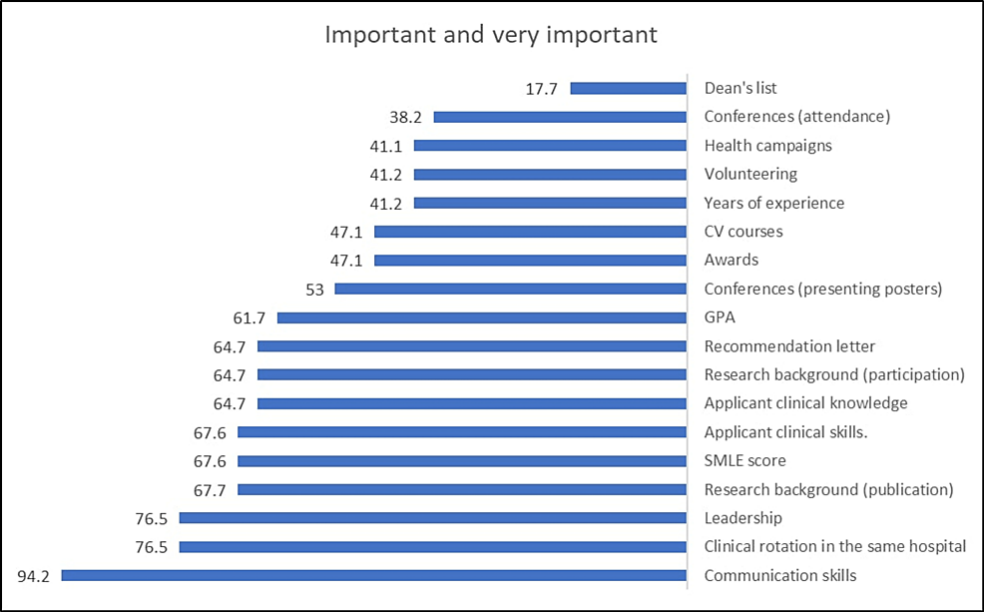
Important and very important selection criteria for GS and INTMED residency programs GS: General surgery; INTMED: Internal medicine.

Figure 1 demonstrates that the most common selection criteria rated as important and very important were communication skills (94.2%), clinical rotation in the same hospital (76.5%), and leadership (76.5%).

**Figure 2:**
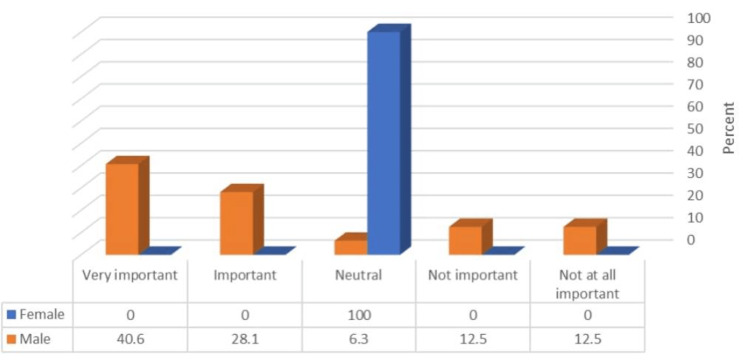
Relationship between the participants’ gender and rating of the importance of the recommendation letter (N = 34) χ^2^ = 15.93, p-value = 0.003.

Figure 2 illustrates that male PDs had a significantly higher rating of the recommendation letter as important and very important (p = <0.05).

**Figure 3:**
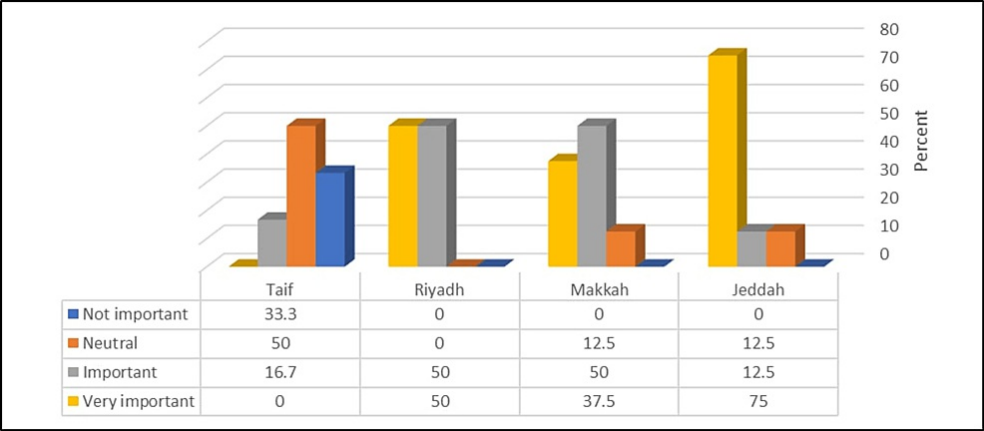
Relationship between the participants’ region and rating of the importance of clinical rotation in the same hospital (electives) (N = 34) χ^2^ = 23.11, p-value = 0.006

Figure 3 shows that PDs in the Riyadh region had a significantly higher rating of the clinical rotation in the same hospital (electives) as very important (p = <0.05).

**Figure 4:**
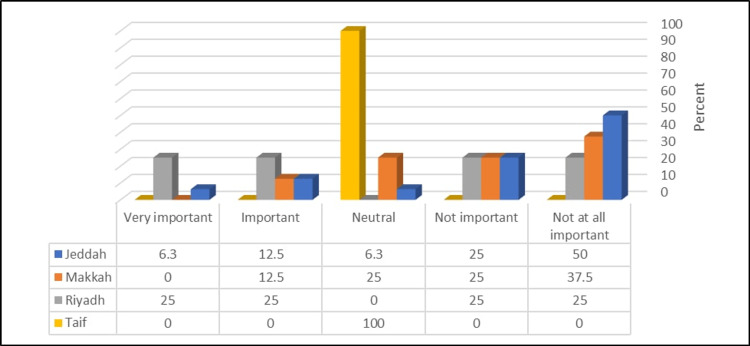
Relationship between the participants’ region and rating of the importance of the dean’s list (N = 34) χ^2^ = 25.14, p-value = 0.014.

Figure 4 shows that PDs in the Jeddah region had a significantly higher rating of the dean's list as very important (p = <0.05).

Table 2 shows that PDs who rated grade point average (GPA) as very important had a significantly lower mean age (p = <0.05), while those who rated the recommendation letter as very important had a significantly higher mean age (p = <0.05).

**Table 2:** Relationship between the participants’ age and rating of the importance of GPA and the recommendation letter (N = 34) * indicates one-way ANOVA. GPA: Grade point average.

Variables	Age					Test* (p-value)
	Not at all important	Not important	Neutral	Important	Very important	
	Mean SD					
GPA	43.33 ± 4.61	41 ± 4.58	39.14 ± 4.52	45.69 ± 4.83	40.63 ± 3.88	3.03 (0.033)
Recommendation letter	42.75 ± 4.71	43.75 ± 6.65	38.75 ± 2.5	39.22 ± 2.63	45.54 ± 4.9	3.59 (0.017)

## Discussion

The present work revealed that the most common selection criteria rated as important were communication skills, clinical rotation in the same hospital, and leadership, while a recommendation letter was ranked the fourth most important criterion.

Cullen et al.’s study of INTMED residents at the Mayo Clinic revealed that professionalism is among the selection criteria [11]. This result is in line with our finding that communication skills were reported as the most important selection criterion.

A cross-sectional study in 2021 of all current and previous plastic surgery training PDs in Saudi Arabia noted the high relevance of leadership, clinical rotation in the same hospital, and communication skills. In this survey, the majority of PDs regarded the recommendation’s mode of communication such as phone calls as the most crucial component. They considered high-quality publications as more highly preferred applicants who had completed electives or rotations in their department [12].

Rating GPA as an important selection criterion was not found in the present study. This could be explained by the reliance on the results of the SMLE. Moreover, although a Jordanian study in 2020 found that a GPA above 80% increased the likelihood of matching with the top two requested specialties, an entry exam score above 70% was the main determining factor [13]. Mardan et al.’s Saudi study found that research experience is the most important selection criterion. This goes against our study in which communication skills, clinical rotation, and leadership were the most important criteria [12].

In the present study, we did not assess the screening of applicants on social media. A previous Saudi study in 2019 examined 41 hospitals involved in Saudi board family medicine training programs. The study found that the percentage of positive attitudes toward ranking applicants using their social media to assess attitudes before interviews and recruitment of potential employees was 55.5% ± 17.3%. The average percentage of positive attitudes was higher for those who used social media to communicate with residents than those who did not use social media even after adjusting for familiarity with internet use. In this study, PDs considered establishing culturally acceptable guidelines to be important for the assessment of e-professionalism and social reputation [14].

## Conclusions

This study investigated PDs’ commonly used selection criteria for GS and INTMED residency programs and identified how those criteria were valued by PDs in the western and central regions of Saudi Arabia. It showed that the most selected criteria for both specialties were communication skills followed by clinical rotation in the same hospital; leadership skills were also highly valued by PDs. By contrast, the dean’s list was given the least consideration according to PDs’ choices. However, applicants should be aware that all the criteria mentioned in this study are valuable and must be considered when applying and preparing for both programs. Nevertheless, further studies might be helpful for determining the selection criteria for other specialties as well as other geographical locations. The main limitation of this study is its small sample size with an unequal number of PDs from both specialties.
